# Seasonal Variability in Airborne Biotic Contaminants in Swine Confinement Buildings

**DOI:** 10.1371/journal.pone.0112897

**Published:** 2014-11-13

**Authors:** Priyanka Kumari, Hong L. Choi

**Affiliations:** Department of Agricultural Biotechnology, Research Institute for Agriculture and Life Science, Seoul National University, Gwanak, Seoul, Republic of Korea; Argonne National Laboratory, United States of America

## Abstract

Little is known about the seasonal dynamics of biotic contaminants in swine confinement buildings (SCBs). The biotic contaminants of seven SCBs were monitored during one visit in the winter and one during the summer. Paired-end Illumina sequencing of the 16S rRNA gene, V3 region, was used to examine seasonal shifts in bacterial community composition and diversity. The abundances of 16S rRNA genes and six tetracycline resistance genes (*tet*B, *tet*H, *tet*Z, *tet*O, *tet*Q, and *tet*W) were also quantified using real-time PCR. Bacterial abundances, community composition and diversity all showed strong seasonal patterns defined by winter peaks in abundance and diversity. Microclimatic variables of SCBs, particularly air speed, PM2.5 and total suspended particles (TSP) were found significantly correlated to abundances, community composition, and diversity of bacterial bioaerosols. Seasonal fluctuations were also observed for four tetracycline resistance genes, *tet*H, *tet*O, *tet*Q, and *tet*W. The frequency of occurrences of these resistance genes were significantly higher in samples collected during winter and was also significantly correlated with air speed, PM2.5 and TSP. Overall, our results indicate that biotic contaminants in SCBs exhibit seasonal trends, and these could be associated with the microclimatic variables of SCBs. The correlations established in the current study could be helpful in establishing better management strategies to minimize the potential health impacts on both livestock and humans working in this environment.

## Introduction

The intensification of pig farming in confined buildings with high animal densities can lead to poor indoor air quality. Microbial decomposition of proteinaceous waste products in feces and urine results in elevated concentrations of volatile organic compounds, ammonia (NH_3_), and sulfides [1], whereas feed materials, skin debris, bedding material, and dried manure generate airborne particulates that carry adsorbed microorganisms and endotoxins [2]. Poor indoor air quality in swine confinement buildings (SCBs) affects both animal and human health. For example, airborne particulates can deposit in nasal channels and the respiratory tract and cause damage to lung tissues [3]. Furthermore, some airborne bacteria and gases such as NH_3_, hydrogen sulphide (H_2_S) (from the manure), and carbon di-oxide (CO_2_) (pig activity) can cause or trigger chronic respiratory tract inflammation in workers and pigs [4]–[11].

Microclimatic variables can influence the formation of aerosols containing microorganisms. There are great variations in the outside temperature in South Korea (−7 to 1°C in winter and from 22°C to 30°C in summer) [12], so maintaining an optimal indoor temperature in SCBs can be challenging. Typically in the winter, all of the openings are closed, and the ventilation rate has to be minimal to reduce the heat loss. This low ventilation rate could induce an increased concentration of airborne contaminants. In contrast, the ventilation is maximal during the summer, thus diminishing the indoor temperature and contributing to driving the indoor air outside the SCBs.

Bacteria constitute a huge proportion of organisms within bioaerosols in SCBs, with a mean concentration of 10^5^ cfu m^−3^ [13], [14]. While much has been done to monitor the indoor airborne biotic contaminants in SCBs [14]–[16], relatively little is known about the seasonal dynamics of airborne biotic contaminants and their interaction with microclimate parameters in SCBs. Nehme *et al.* [14], [15] examined the seasonal variability of airborne bacterial and archaeal communities in SCBs and found that, although the microbial abundances were significantly higher during the winter, the biodiversity was similar in each SCB during both the winter and summer seasons. However, these studies used low-resolution molecular fingerprinting tools, which lacked the coverage and depth of high-throughput sequencing methods. In a recent study, Hong et al. [16] used 454-pyrosequencing to analyze the airborne biotic contaminants in pig and poultry confinement buildings sampled from different climate conditions and found that the different livestock as well as production phase were associated with distinct airborne bacterial communities; however, they did not evaluate the effect of microclimate variables on bacterial bioaerosol communities.

The usage of antibiotics in swine farms has promoted the development and abundance of antibiotic resistance in microbes [17], [18], which can become aerosolized within the SCBs. Antibiotic resistant genes (ARGs) can be transferred to pathogens through transformation or phage-mediated transduction, and/or by conjugation, posing a serious threat to public health. Horizontal gene transfer plays important roles in the evolution and transmission of ARGs between bacterial species and includes the movement of ARGs from fecal bacteria to environmental bacteria, as well as the reverse; that is, emergence of novel mechanisms of acquired resistance in pathogens, ARGs that originally were present in harmless bacteria [19]. Tetracycline was chosen for this study because it is the most widely used broad spectrum antibiotic in livestock production worldwide, and is particularly prevalent in pig production [20]. The mechanism by which bacteria resist tetracycline antibiotics is heavily biased by ecological niche [21], and compared to the existing literature on tetracycline resistance genes (Tc^R^) in soils and in water, relatively little is known regarding Tc^R^ genes in aerosols of SCBs [16], [22]. Three Tc^R^ genes (*tet*B, *tet*H, *tet*Z) encoding efflux proteins (EFP), and three others (*tet*O, *tet*Q, *tet*W) encoding ribosomal protection proteins (RPP) were selected for this study because these genes have been detected in aerosol of SCBs [16] and because these Tc^R^ genes encode two main mechanisms of bacterial resistance to tetracycline, which have been found associated with bacteria of public health interest [23]–[25].

Therefore, the aim of this study was to answer the following questions using the Illumina Hiseq sequencing of the V3 region of the 16S rRNA gene and qPCR of both 16S rRNA and Tc^R^ genes: (1) How does the bacterial bioaerosol community composition and diversity vary in SCBs during the winter and summer seasons? (2) Does the abundance of 16S rRNA and Tc^R^ genes vary in SCBs during the winter and summer seasons? (3) What are the major microclimate variables affecting the abundance, community composition, and diversity of airborne biotic contaminants in SCBs?

## Materials and Methods

### Characteristics of animal confinement buildings

The study was conducted in the winter (January) and summer (June) of 2013 on seven commercial pig farms located in six South Korean provinces. All the commercial pig farms sampled in this study are privately owned. Permission to access privately owned farms was given by the farm owners and for future permissions we can contact the owners. All samples were collected in the growing/finishing houses of SCBs. The average outdoor temperature across all of South Korea ranges from −7 to 1°C in winter and 22°C to 30°C in summer. Temperature differences among the six provinces were less than 2°C [12]. Ventilation in SCBs was mechanical by exhaust fans on walls. The number of animals kept in each sampling room ranged from 140 to 480, and the stocking density varied from 0.88 to 1.41 m^2^/head. The age of the pigs varied from 67–150 days in each sampling room at the time of sampling. The manure removal system was deep-pit with slats, and the cleaning cycle varied from 4–6 months.

### Microclimate variables

The microclimate variables were measured from three points in the aisle outside the pens at every 8 h interval till 24 h (Fig. S1), and all the microclimate variables were reported as averages corresponding to each sampling period. Air temperature and relative humidity were measured with a hygrothermograph (SK-110TRH, SATO, Tokyo, Japan). Air speed was measured with an anemometer (model 6112, KANOMAX, Osaka, Japan). Particulate matter concentrations (µg m^−3^) were measured using an aerosol mass monitor (GT-331, SIBATA, Soca-city, Japan). The mass concentrations of PM10 (PM average aerodynamic diameter ≤10 µm), PM2.5 (PM mean aerodynamic diameter ≤2.5 µm), PM1 (PM mean aerodynamic diameter ≤1 µm), and total suspended particles (TSP) were obtained simultaneously, at a flow rate of 2.83 l min^−1^. The concentrations of NH_3_, H_2_S and CO_2_ were measured by gas detector tubes (Gastec Co., Ltd., Kanagawa, Japan).

### Sample collection and DNA extraction

Aerosol samples were collected from the middle point in the aisle outside the pens at a height of 1.4 m above the floor (Fig. S1). Air samples were captured on sterile 0.22-µm cellulose nitrate filters (Fisher Scientific, Pittsburgh, PA) via vacuum filtration with a flow rate of 4 l min^−1^ for 24 h. The cellulose nitrate filters were kept at 4°C until processing in the laboratory. Bacterial DNA was extracted directly from the filters using the PowerSoil DNA isolation kit (MoBio Laboratories, Carlsbad, CA). Individual filters were aseptically cut into small pieces, loaded into the bead tube of the DNA extraction kit, and heated to 65°C for 10 min followed by 2 min of vortexing. The remaining steps of the DNA extraction were performed according to the manufacturer's instructions. The purified DNA was resuspended in 50 µl of solution S6 (MoBio Laboratories) and stored at −20°C until PCR amplification. Blank filters were analyzed alongside sample filters to test for contamination, and following DNA extraction and amplification, blank filters were consistently found to be free of microbial contaminants.

### Illumina sequencing and data processing

The extracted DNA was amplified using primers 338F (5′-XXXXXXXX-GTACTCCTACGGGAGGCAGCAG-3′) and 533R (5′-TTACCGCGGCTGCTGGCAC-3′) targeting the V3 region of bacterial 16S rRNA (‘X’ denotes 8-mer barcode sequence) [26]. Paired-end sequencing was performed at Beijing Genome Institute (BGI) (Hongkong, China) using 2×150 bp Hiseq2500 (Illumina) according to the manufacturer's instructions. Library preparation, sequencing, and initial quality filtering were performed as described previously [27]. The sequence data obtained by Illumina Hiseq2500 sequencing were processed using mothur [28]. Paired-end sequences were assembled, trimmed, and filtered in mothur. Next, the sequences were aligned against a SILVA alignment (http://www.arb-silva.de/). Putative chimeric sequences were detected and removed via the Chimera Uchime algorithm contained within mothur [29]. Sequences were denoised using the ‘*pre.cluster*’ command in mothur, which applies a pseudo-single linkage algorithm with the goal of removing sequences that are likely due to sequencing errors [30]. All sequences were classified using the EzTaxon-e database (http://eztaxon-e.ezbiocloud.net/) [31], using the classify command in mothur at 80% Naïve Bayesian bootstrap cutoff with 1000 iterations. Sequence data were deposited in SRA at NCBI with the accession number of SRP039383.

### Quantification of 16S rRNA and Tc^R^ genes

A real-time polymerase chain reaction was used to quantify bacterial 16S rRNA and six Tc^R^ genes (RPP class: *tet*O, *tet*Q and *tet*W; EFP class: *tet*B, *tet*H and *tet*Z, refer Levy et al. [32] for the details on nomenclature) using the SYBR Green approach with the primers described in Table S1. The copy numbers of 16S rRNA and Tc^R^ genes in bioaerosol samples were measured against the standard curves of plasmids containing copies of the respective genes, using a 10-fold serial dilution. All reactions were conducted in triplicate with the 20 µl qPCR mixtures containing 10 µl of 2× SYBR Green PCR Master Mix (Applied BioSystems, Foster City, CA, USA), 1.0 µl each of the 10 µM forward and reverse primers, and 7.0 µl of sterile, DNA-free water. Standard and bioaerosol (ca. 1.0 ng) DNA samples were added at 1.0 µl per reaction. The reaction was carried out on an ABI Prism 7300 sequence detector (Applied Biosystems, Foster City, CA, USA) with an initial step of 95°C for 10 min, followed by 40 cycles of denaturation (95°C for 10 s), and primer annealing and extension (60°C for 1 min). Dissociation curve analysis was performed to ensure the specificity of PCR, which included an increment of temperature from 60°C to 95°C at an interval of 0.5°C for 5 s. Gene copy numbers were determined using a regression equation for each assay and relating the cycle threshold (CT) value to the known numbers of copies in the standards. The correlation coefficients of standard curves ranged from 0.957 to 0.996.

### Statistical processing and analysis of results

Rarefaction curves and diversity indices were generated using mothur, with the bacterial phylotype (OTU) defined here at 97% threshold of 16S rRNA gene sequence similarity. Phylogenetic diversity (PD) was calculated as Faith's PD [33], the total phylogenetic branch length separating OTUs in each rarefied sample. To allow for robust comparisons among samples containing different numbers of sequences, phylotype richness and phylogenetic diversity were calculated based on samples rarefied to contain 15,909 sequences. To test for differences in relation to season on OTU richness, PD, 16S rRNA and Tc^R^ genes abundances, we used a t-test for normal data and the Wilcoxon rank-sum test for non-normal data. We used the same procedure to test whether the relative abundance of the most abundant phyla differed across seasons.

To avoid including collinear variables in further analyses, we used a Spearman's correlation matrix and highly correlated (Spearman's *r*≥0.8) microclimatic variables were removed from further analysis. Non-metric multidimensional scaling (NMDS) was generated based on pairwise Bray-Curtis dissimilarities between samples using the vegan R package [34]. The analysis of similarity (ANOSIM) function in the vegan R package with 999 permutations was used to test for differences in bacterial communities among the winter and summer season. The vectors of microclimate variables were fitted onto ordination space (Bray–Curtis NMDS) to detect possible associations between patterns of community structure and microclimate variables using the ‘envfit’ function of the vegan R package, and statistical significance was evaluated among 999 random permutations. Analyses for Venn diagram generation were performed using the mothur, and the Venn diagram was plotted using *R* package VennDiagram [35]. Differentially abundant bacterial genera between the winter and summer seasons were identified using a parametric approach (Metastats) [36]. All statistical analysis, graphs, and ordinations were produced using R version 3.0.2 [37].

## Results

The means of microclimate variables in the SCBs during the winter and summer seasons are presented in Table 1. All of the microclimate variables in the SCBs differed significantly between the winter and summer season samples (*P*<0.05; Table 1), except total suspended particles, NH_3_ and H_2_S (*P*>0.05, Table 1). Spearman's correlation matrix showed highest correlation between PM10 and TSP (*r* = 0.93; Table 2). Temperature and airspeed were the next most correlated variables (*r* = 0.9; Table 2) followed by NH_3_ and CO_2_ (r = 0.82; Table 2) and PM2.5 and PM1 (r = 0.81; Table 2). Therefore, we removed PM10, temperature, PM1 and NH_3_, and used the remaining six microclimate variables, (i.e. airspeed, relative humidity, PM2.5, TSP, H_2_S and CO_2_) for further analyses.

**Table 1:** Seasonal means (±SD) of microclimate variables in swine confinement buildings.

Variable	Temperature, °C	Relative humidity, %	Air speed, m/s	PM10, µg m^−3^	PM2.5, µg m^−3^	PM1, µg m^−3^	TSP, µg m^−3^	NH_3_, mg/L	H_2_S, mg/L	CO_2_, mg/L
Season										
Winter	20.8±5.5	68.5±18.6	0.02±0.02	470.8±468.8	48.4±27	25.7±20.1	1252.2±1320.5	24.4±22.7	0.36±0.34	2833.1±1433.6
Summer	31.5±4.2	86.3±13.7	0.18±0.06	77.5±19.5	17.1±8.2	7.6±4.2	130.8±34.2	14.9±12.1	0.41±0.43	1242.6±423.7
*P*-value^a^	<0.001	0.003	<0.001	0.04	0.03	0.03	0.07	0.21	0.82	0.03

a For each variable, *P*-value was used to determine the significance of means among the two seasons.

**Table 2:** Spearman rank correlations between measured microclimatic variables in SCBs.

Microclimate variables	Temperature	Relative humidity	Air speed	PM10	PM2.5	PM1	TSP	NH_3_	H_2_S	CO_2_
Temperature	1									
Relative humidity	0.26	1								
Air speed	0.9***	0.3	1							
PM10	−0.52	−0.46	−0.39	1						
PM2.5	−0.69**	−0.35	−0.7**	0.72**	1					
PM1	−0.63*	−0.17	−0.64*	0.34	0.81***	1				
TSP	−0.39	−0.48	−0.23	0.93***	0.53	0.18	1			
NH_3_	−0.06	0.02	−0.28	−0.13	0.12	−0.02	−0.15	1		
H_2_S	0.21	−0.11	−0.06	−0.11	0.04	−0.11	−0.15	0.68**	1	
CO_2_	−0.26	−0.02	−0.46	0.19	0.48	0.3	0.14	0.82***	0.44	1

*P<0.05;

**P<0.01;

***P<0.001.

**Figure 1:**
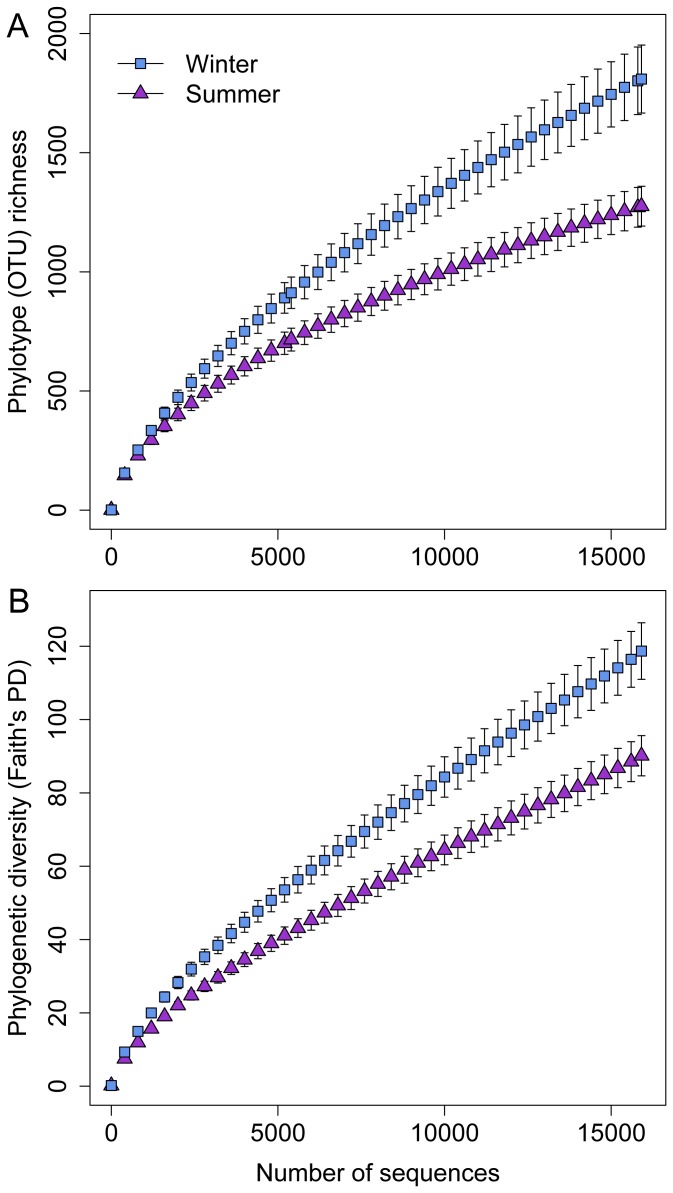
Rarefaction curves describing the bacterial OTU richness (A) and phylogenetic diversity observed in the bioaerosol of SCBs during the winter and summer seasons. Diversity indices were calculated using random selections of 15,909 sequences per sample and error bars represent +1 s.e.m.

From the 14 samples, we obtained 13,597 OTUs at 97% similarity from 497,607 good-quality sequences. The average number of OTUs per sample was 2442±910 (standard deviation [SD]), ranging from 1287 to 4045 OTUs. To compare diversity levels and community profiles between samples controlling for differences in sequencing depth, samples were compared at the same sequencing depth (15,909 randomly selected sequences per sample). At this depth of coverage, bacterial richness (*P*<0.01; Fig. 1A) and phylogenetic diversity levels (*P* = 0.01; Fig. 1B) were significantly higher in winter in comparison to summer. Spearman's correlation coefficients showed a significant negative correlation between air speed and both OTU richness and PD of bacterial bioaerosol communities (Table 3). Whereas, PM2.5 and TSP were positively correlated to OTU richness and PD (Table 3).

**Table 3:** Spearman rank correlations between microclimatic variables, diversity and abundances of airborne biotic contaminants in SCBs.

Microclimate variables	OTU richness	Phylogenetic diversity	16S rRNA copy number	*tet*B	*tet*H	*tet*Z	*tet*O	*tet*Q	*tet*W
Relative humidity	−0.56*	−0.52	−0.4	−0.24	−0.26	−0.24	−0.47	−0.3	−0.39
Air speed	−0.7**	−0.71**	−0.78***	−0.14	−0.69**	−0.52	−0.61*	−0.68**	−0.73**
PM2.5	0.57*	0.57*	0.78***	0.24	0.62*	0.66*	0.58*	0.79***	0.79***
TSP	0.08	0.06	0.55*	0.45	0.56*	0.57*	0.64*	0.58*	0.6*
H_2_S	0.35	0.31	0.09	−0.11	−0.25	0.1	−0.19	−0.03	−0.07
CO_2_	0.42	0.4	0.56*	−0.24	0.32	0.63*	0.41	0.6*	0.49

*P<0.05;

**P<0.01;

***P<0.001.

**Figure 2:**
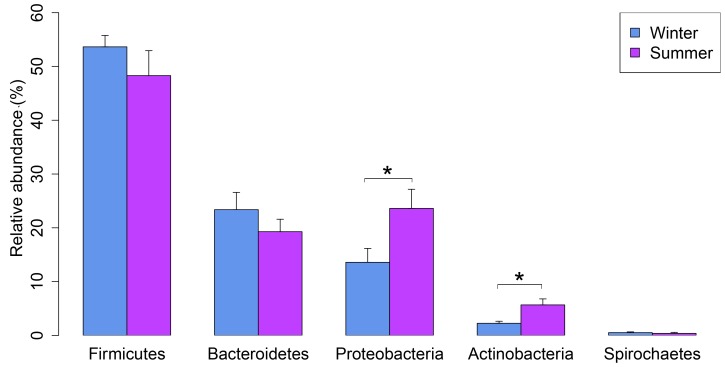
Relative abundance (means ± SE) of the most abundant bacterial phyla detected in SCBs bioaerosol during the winter and summer seasons. * indicates significantly different at >0.05.

**Figure 3:**
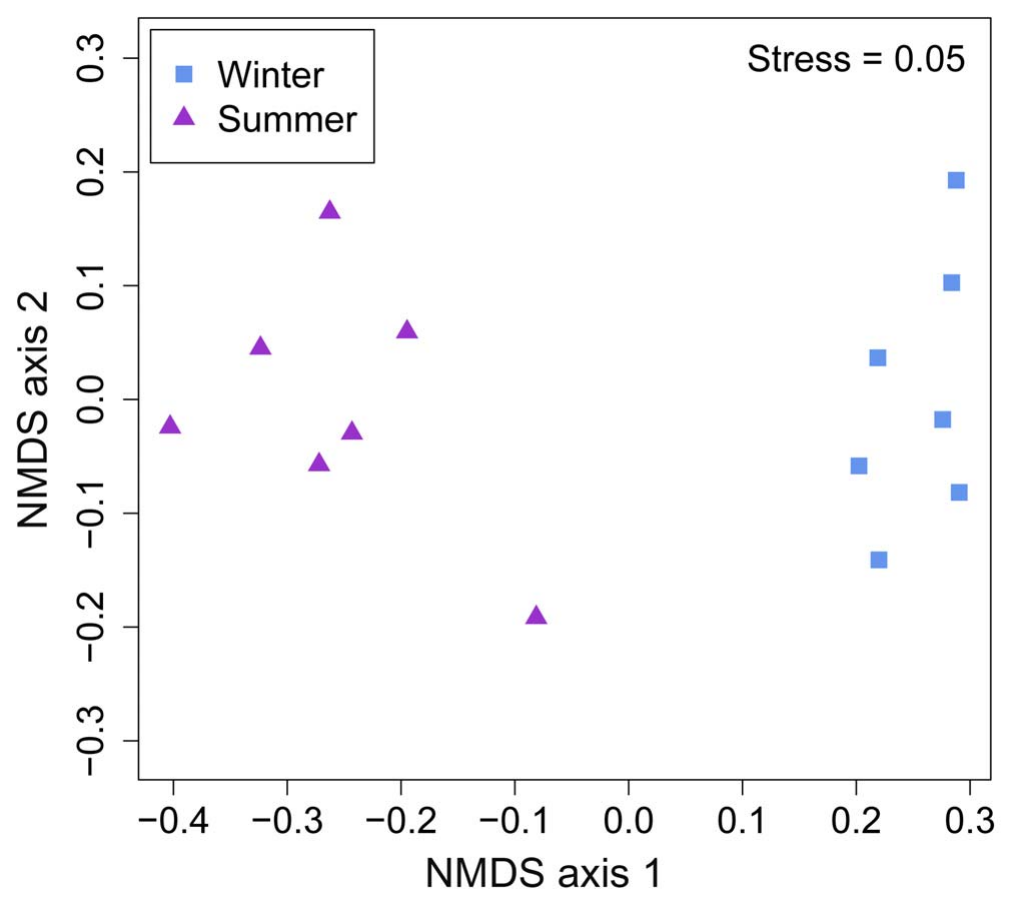
NMDS of Bray-Curtis pairwise dissimilarity of bacterial bioaerosol community in SCBs during the winter and summer seasons.

The most abundant bacterial phyla were *Firmicutes*, representing 50.9% of all sequences, followed by *Bacteroidetes* (21.3%), *Proteobacteria* (18.5%), and, to a lesser degree, *Actinobacteria* (3.9%), *Tenericutes* (0.6%), and *Spirochaetes* (0.4%); 0.8% of all sequences were unclassified. We found significant differences in relative abundance across seasons for *Proteobacteria* (*P* = 0.04) (Fig. 2) and *Actinobacteria* (*P* = 0.03) (Fig. 2). The composition of the airborne bacterial communities was significantly influenced by seasons (ANOSIM statistic R = 0.96, *P*<0.01; Fig. 3). The samples collected during the winter season harbored bacterial communities distinct from those found in samples collected during summer. An environmental fitting analysis, using microclimatic variables, showed that airspeed (*r* ^2^ = 0.70, *P* = 0.002), PM2.5 (*r* ^2^ = 0.39, *P* = 0.04), TSP (*r* ^2^ = 0.39, *P* = 0.04) and CO_2_ (*r* ^2^ = 0.43, *P* = 0.04) were significantly associated with bacterial community composition.

Venn diagrams (Fig. S2) illustrate that OTU overlap between seasons as well as show unique OTUs. Overall, 20% of OTUs were shared between the winter and summer season. These 2,705 shared OTUs represented the majority of sequences (457,100 sequences or 91% of the total data set) (Fig. S2). Given the distinct clustering pattern in bacterial community composition in SCBs by season, we investigated in more detail what bacterial genera determine more strongly the distinct community composition in each season. Some bacterial genera were found to be dominant in both winter and summer seasons. *Lactobacillus* (18.3% and 16.3% on average) and *Prevotella* (19.6% and 6.2%) were the most dominant genera in both seasons. However, there were some differences in abundant genera between the two seasons (Table 4). The bacterial bioaerosol of SCB in the winter season was dominated by a single genus – *Prevotella* – at nearly 19.6% (Metastats *P* = 0.01). *Faecalibacterium* (0.8%), *Blutia* (0.7%), and *Catenibacterium* (0.7%) were also more abundant in the winter than in the summer (all *P*≤0.01). Genera that were more abundant in the summer included *Sphingomonas* (3.7%), *Capnocytophaga* (3%), *Haemophilus* (2.8%), and *Streptococcus* (2.6%) (all *P*≤0.01).

**Table 4:** Differentially abundant bacterial genera in swine confinement buildings sampled during winter and summer seasons.

Phylum	Genus	Winter (%)	Summer (%)	p-value
*Bacteroidetes*	*Capnocytophaga*	0.00	**3.06**	<0.01
*Bacteroidetes*	*Chitinophaga*	0.01	**0.68**	<0.01
*Bacteroidetes*	*Dyadobacter*	0.00	**0.37**	<0.01
*Bacteroidetes*	*Prevotella*	**19.57**	6.24	0.01
*Firmicutes*	*Blautia*	**0.75**	0.19	0.01
*Firmicutes*	*Bulleidia*	**0.56**	0.33	<0.01
*Firmicutes*	*Butyricicoccus*	**0.49**	0.19	0.01
*Firmicutes*	*Catenibacterium*	**0.74**	0.28	<0.01
*Firmicutes*	*Faecalibacterium*	**0.87**	0.20	<0.01
*Firmicutes*	*Oscillibacter*	**0.38**	0.12	0.01
*Firmicutes*	*Ruminococcus*	**0.33**	0.11	<0.01
*Firmicutes*	*Streptococcus*	0.37	**2.64**	0.01
*Firmicutes*	*Subdoligranulum*	**0.69**	0.28	0.01
*Proteobacteria*	*Escherichia*	0.02	**1.26**	<0.01
*Proteobacteria*	*Haemophilus*	0.00	**2.82**	<0.01
*Proteobacteria*	*Neisseria*	0.00	**0.32**	0.01
*Proteobacteria*	*Sphingomonas*	0.17	**3.70**	0.01

Differences in relative abundance of bacterial genera between seasons are represented with Metastats p-values. Significantly different (*P*≤0.01) and relatively abundant genera (>0.3%) were displayed.

**Figure 4:**
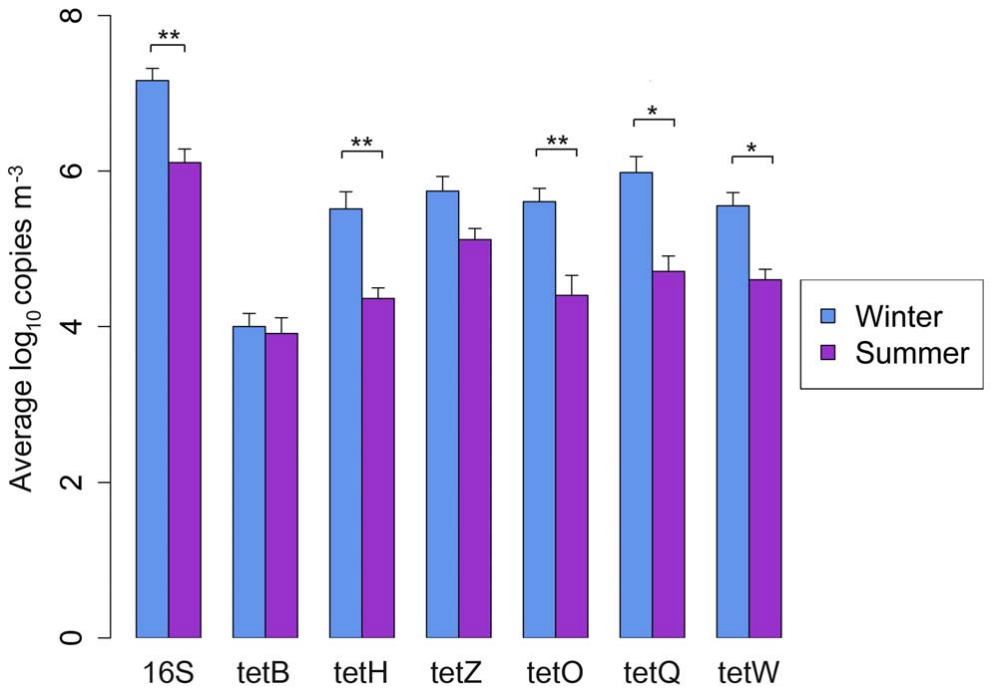
Abundance of 16S rRNA and tetracycline resistance genes in the bioaerosols of SCBs. Asterisks above solid lines indicate significant differences between the winter and summer seasons samples of SCBs. * indicates significantly different at >0.05, ** indicates at >0.01.

The abundances of 16S rRNA genes in the bioaerosols of SCBs were significantly higher in the winter (mean = 1.4×10^8^ bacteria m^−3^, *n* = 7, *P*<0.01, Fig. 4) than in the summer (mean = 1.2×10^7^ bacteria m^−3^, *P*<0.01, *n* = 7, Fig. 4). Six classes of Tc^R^ genes (*tet*B, *tet*H, *tet*Z, *tet*O, *tet*Q, and *tet*W) were further quantified using qPCR. All six classes of Tc^R^ genes were detected in high abundance in both winter and summer bioaerosol samples (Fig. 4). Tc^R^ genes encoding RPP (*tet*O, *tet*Q and *te*tW) were present in significantly higher copy numbers than Tc^R^ encoding EFP (*tet*B, *tet*H and *tet*Z; *t*-test, *P*-value = 0.04). Seasonal trends were also detected in four Tc^R^ genes, which include *tet*H, *tet*O, *tet*Q, and *tet*W, and their abundances peaked in bioaerosol samples collected during the winter (Fig. 4). Among the six microclimate variables, air speed was found negatively correlated (*P*<0.05) with the abundances of 16S rRNA, *tet*H, *tet*O, *tet*Q, and *tet*W genes (Table 3), however, PM2.5 and TSP were found positively correlated (*P*<0.05) with these genes (Table 3).

## Discussion

In this study, Illumina sequencing was used to provide a comprehensive insight into the bacterial bioaerosol community composition and diversity in SCBs. The use of high-throughput molecular sequencing methods revealed indoor microbial biodiversity that was previously difficult or impossible to observe [38].

Our results indicate that bacterial bioaerosol diversity was significantly higher in winter and this result is in contrast with the findings of Nehme et al. [14], who examined the influence of seasonal variation on bacterial biodiversity in SCBs and found that biodiversity was unchanged between different seasons of the year. One of the possible explanations for this discrepancy in results could be that Nehme et al. [14] analyzed very limited number of sequences for estimating the bacterial bioaerosol diversity. Furthermore, Nehme et al. [14] used denaturing gradient gel electrophoresis and 16S-cloning-and-sequencing approach to characterize the bacterial bioaerosol community, which lack resolution and throughput, respectively, compared to next-generation sequencing based methods [39]–[41]. We found that both air speed and particulate matter strongly influenced the bacterial bioaerosol diversity. Airspeed has been shown to impact the diversity of indoor bacterial communities [42], and these results suggest that the higher diversity levels in the winter season are likely to be a function of ventilation rate and particulate matter, as during summer under high ventilation rates, more airborne particulate matter carrying bacteria would be transferred out of the SCBs.

Seasonality also influenced bacterial bioaerosol community composition and most of the observed variation was explained by the microclimate variables. In several previous studies, it has been reported that microclimate variables are the most important factor which affects the indoor bacterial bioaerosol community composition and diversity [42], [43]. This relationship could be due to a direct link between the growth and survival of certain taxa and microclimate conditions in SCBs, or an increase in the dispersal of microbes from animals or animal feces under these conditions. Consistent with previous studies [14], [16], [44], our bioaerosol samples of SCBs were dominated by the phyla *Firmicutes*, *Bacteroidetes*, *Proteobacteria*, and *Actinobacteria*. The genera *Lactobacillus* and *Prevotella*, which accounted for the predominant bacterial genera in our samples, are commonly associated with the fecal microbiota; this result further validates the claims that swine feces and soiled bedding material are the primary source of the bacteria in the bioaerosols of SCBs [14], [16], [45]. Although many of the genera described were detected in both seasons, many others were only detected in one of the seasons, thus justifying the view that the bacterial bioaerosol communities in both the winter and summer season samples are different.

The high abundance of 16S rRNA and Tc^R^ genes detected in the bioaerosol samples suggests an alternative airborne transmission route, which can lead to their persistence and dispersal into the nearby environment. The level of contamination detected in this study was far higher than the proposed limit of bacterial contamination associated with human respiratory symptoms [8]. Similar to several previous studies, our results indicate that swine workers are exposed to a higher level of airborne bacteria than the occupational recommendations [13],[14],[44]. The prevalence of Tc^R^ genes encode RPP in the present study is not surprising, since these genes were found to be predominant in the gastrointestinal tracts of pigs and steers [46], and their elevated possibilities of transfer from one bacteria to another because of their close relationship with mobile genetic elements such as plasmids, conjugative transposons, integrons, and consequently their wide host range [23], [24]. Among all Tc^R^ genes, *tet*Q had the highest abundance in bioaersol samples (the average abundance was 8.89×10^5^±1.45×10^6^ copies m^−3^) followed by *tet*Z, *tet*O, *tet*W and *tet*H, with *tet*B having the least abundance. The relatively high level of *tet*Q is not surprising because it is seen equally in both Gram-positive and Gram-negative bacterial genera [23], and most of which have been shown to dominate the bioaerosols of SCBs such as *Clostridium*, *Lactobacillus*, *Staphylococcus*, *Streptococcus*, *Prevotella* etc. [14], [16], [44]. The lack of *tet*B and *tet*H is also not surprising because they are found only associated with Gram-negative bacteria [23], which are less common in bioaerosols of SCBs. However, higher average abundance of *tetZ* compared to *tet*O and *tet*W was not expected because it has relatively narrow host range (Only detected in *Lactobacillus* and *Corynebacterium*; [23]). These results are consistent with the recent findings that both ecology and bacterial phylogeny are the primary determinant of ARG content in environment [21], [47]. qPCR is more indicative of the potential for aerosol-mediated transfer of antibiotic resistance between environments than culture-based methods, and results can be more easily compared among studies. Nonetheless, the method is limited by it its ability to detect only a fragment of genes targeted. Truncated sequences and non-expressed sequences cannot be resolved from expressed gene sequences using qPCR, so the levels reported could overestimate the number of functional, full length genes.

Our findings also revealed that 16S rRNA and most of the Tc^R^ genes showed higher abundance in winter bioaerosol samples. Seasonal trends in microbial 16S rRNA gene concentration has already been reported by Nehme et al. [15], [29], who showed that total microbial 16S rRNA genes concentrations in bioaersol of SCBs were significantly higher in winter. Seasonal fluctuation in Tc^R^ genes abundances have been reported several times in wastewater treatment plants and livestock lagoons [48], [49]; however, this is the first time a seasonal trend has been reported in bioaerosol samples of SCBs. The abundance of these genes were negatively correlated to airspeed and positively correlated to PM2.5 and TSP. The reduced ventilation in SCBs to avoid heat loss in winter could be responsible for the increased concentration of TSP and PM2.5 in SCBs, which in turn could increase the abundance of 16S rRNA and Tc^R^ genes. In this survey only a limited number of ARGs were investigated in bioaersols, however, a variety of ARGs encoding different antibiotic resistance could be present in bioaerosols of SCBs, where different classes of antibiotics (e.g., macrolides, lincosamides) are frequently used in addition to tetracycline. So there is a need for further study to explore more diverse ARGs in bioaersols of SCBs.

The detection of 16S rRNA and Tc^R^ genes in high abundance is of particular concern, because Tc^R^ airborne pathogenic bacteria present in SCBs have been shown to colonize the nasal flora of pig farmers [50], and this could pose potential occupational health problem. Indoor air ventilated from the SCBs to the external environment can cause detrimental effects to the ambient air quality. For instance, multiple drug resistances bacteria were recovered in an air plume up to 150 m downwind from a SCB at higher percentages than upwind [51]. Furthermore, presence of some airborne pathogens have been detected over long distances from their emission site which were found capable to infect healthy animals intramuscularly or intratracheally [52]. Most of the previous studies consistently indicated an association between environmental exposure to SCBs and respiratory symptoms indicative of asthma of their neighbors [53]–[55]. Surprisingly, Smit et al. [56] found an inverse association between indicators of air pollution from livestock farms and respiratory morbidity among neighboring resident. However, before drawing firm conclusions from this study, these results should be confirmed with more objective disease information.

Though, this study had fewer samples than previous studies, our results however indicate that seasons have an influence on the biotic contaminants abundance, community composition and diversity, in indoor air of SCBs. Seasonality was significantly associated with microclimate variables, indicating that indoor environmental conditions play an important role in structuring airborne biotic contaminants in SCBs. Based on the results of this study, better management practices and regulations can be designed to minimize the potential health impact on both the farm workers and the public residing in close proximity to these buildings.

## Supporting Information

Figure S1 **Indoor plan view of sampling points (circles) in swine confinement buildings.** The microclimate variables were measured from 3 points (P1, P2 and P3) and aerosol samples were collected from the middle point (P2) in the aisle outside the pens.
(TIF)

Figure S2 **Venn diagrams showing the overlap of OTUs (at 97% similarity) between winter and summer seasons.** All samples in each season were pooled and then the percentage of shared and season-specific OTUs was calculated.
(TIFF)

Table S1 **Q-PCR primers used to quantify the abundance of 16S rRNA genes and tetracycline resistance genes.**
(DOCX)
