# Supplementary figures and images for: Seasonal Variability in Airborne Biotic Contaminants in Swine Confinement Buildings
Source: PLoS One. 2014 Nov 13;9(11):e112897. doi: 10.1371/journal.pone.0112897 (PMC4231085; doi:10.1371/journal.pone.0112897)

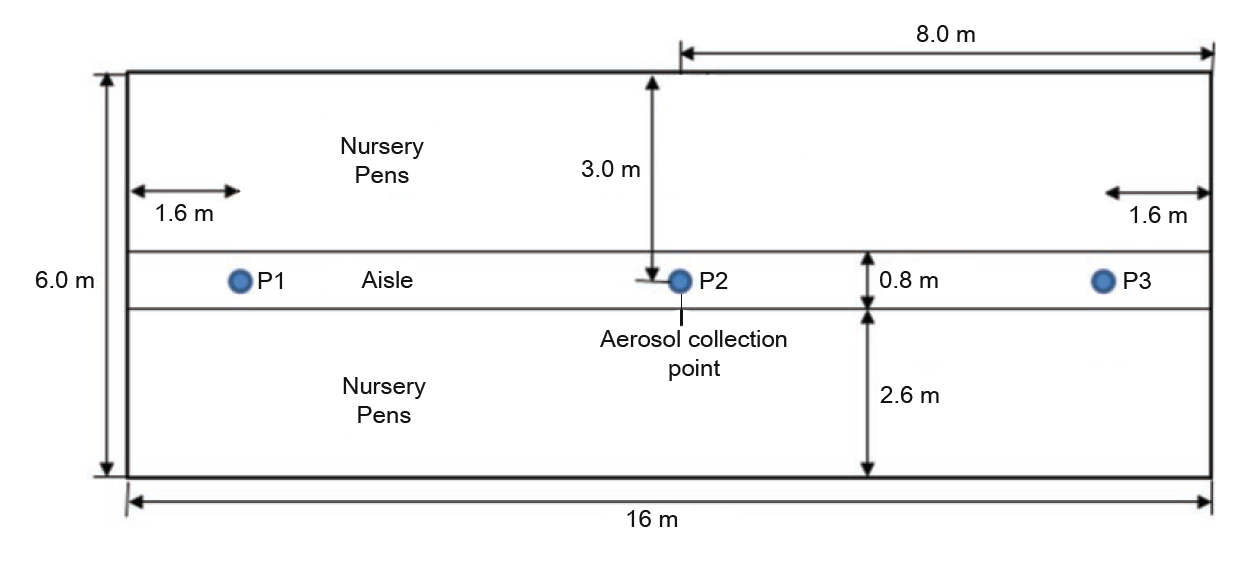

Supplement: Figure S1 — Indoor plan view of sampling points (circles) in swine confinement buildings. The microclimate variables were measured from 3 points (P1, P2 and P3) and aerosol samples were collected from the middle point (P2) in the aisle outside the pens. (TIF) [file pone.0112897.s001.tif]

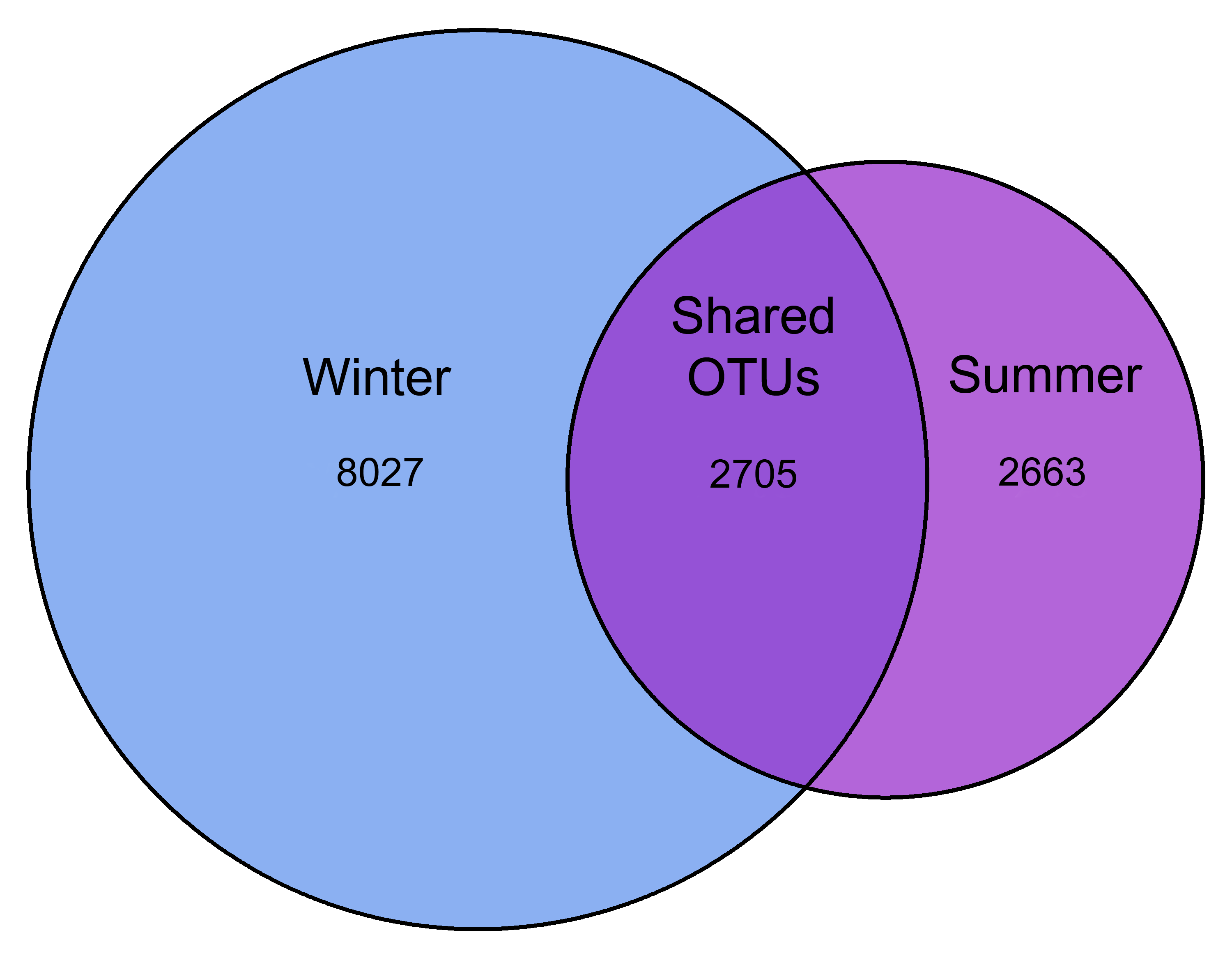

Supplement: Figure S2 — Venn diagrams showing the overlap of OTUs (at 97% similarity) between winter and summer seasons. All samples in each season were pooled and then the percentage of shared and season-specific OTUs was calculated. (TIFF) [file pone.0112897.s002.tiff]
