# Supplementary material for: Seasonal Variability in Airborne Biotic Contaminants in Swine Confinement Buildings
Source: PLoS One. 2014 Nov 13;9(11):e112897. doi: 10.1371/journal.pone.0112897 (PMC4231085; doi:10.1371/journal.pone.0112897)
Supplement: Table S1 — Q-PCR primers used to quantify the abundance of 16S rRNA genes and tetracycline resistance genes. (DOCX) [file pone.0112897.s003.docx]

**Table S1.** Q-PCR primers used to quantify the abundance of 16S rRNA genes and tetracycline resistance genes.

| Primer names | Target gene | Sequence (5’-3’) | Resistance mechanism |
| --- | --- | --- | --- |
| 338F | 16S rRNA gene | ACT CCT ACG GGA GGC AGC | Not applicable |
| 519R |  | GWA TTA CCG CGG CKG |  |
| TetB-FW | *tet*B | TAC GTG AAT TTA TTG CTT CGG | Efflux pumps^1^ |
| TetB-RV |  | ATA CAG CAT CCA AAG CGC AC |  |
| TetH-FW | *tet*H | CAG TGA AAA TTC ACT GGC AAC |  |
| TetH-RV |  | ATC CAA AGT GTG GTT GAG AAT |  |
| TetZ-FW | *tet*Z | CCT TCT CGA CCA GGT CGG |  |
| TetZ-RV |  | ACC CAC AGC GTG TCC GTC |  |
| TetO-FW | *tet*O | ACG GAR AGT TTA TTG TAT ACC | Ribosomal protection proteins^2^ |
| TetO-RV |  | TGG CGT ATC TAT AAT GTT GAC |  |
| TetQ-FW | *tet*Q | AGA ATC TGC TGT TTG CCA GTG |  |
| TetQ-RV |  | CGG AGT GTC AAT GAT ATT GCA |  |
| TetW-FW | *tet*W | GAG AGC CTG CTA TAT GCC AGC |  |
| TetW-RV |  | GGG CGT ATC CAC AAT GTT AAC |  |

**References:**

^1^ Aminov, R.I., Chee-Sanford, J.C., Garrigues, N., Teferedegne, B., Krapac, I.J., White, B.A. and Mackie, R.I. (2002) Development, validation, and application of PCR primers for detection of tetracycline efflux genes of gram-negative bacteria. Appl Environ Microbiol 68: 1786-1793.

^2^ Aminov, R.I., Garrigues-Jeanjean, N., and Mackie, R.I. (2001) Molecular ecology of tetracycline resistance: development and validation of primers for detection of tetracycline resistance genes encoding ribosomal protection proteins. Appl Environ Microbiol 67: 22-32.
